# Efficient Nanocrystal Photovoltaics via Blade Coating Active Layer

**DOI:** 10.3390/nano11061522

**Published:** 2021-06-09

**Authors:** Kening Xiao, Qichuan Huang, Jia Luo, Huansong Tang, Ao Xu, Pu Wang, Hao Ren, Donghuan Qin, Wei Xu, Dan Wang

**Affiliations:** 1 School of Materials Science and Engineering, South China University of Technology, Guangzhou 510640, China; ddkndyx@163.com (K.X.); hqc666666nb@163.com (Q.H.); l5865421014@126.com (J.L.); 201865320283@mail.scut.edu.cn (H.T.); m13856403915@163.com (A.X.); 18145717332@163.com (P.W.); renhns@foxmail.com (H.R.); 2 State Key Laboratory of Luminescent Materials & Devices, Institute of Polymer Optoelectronic Materials & Devices, South China University of Technology, Guangzhou 510640, China

**Keywords:** CdTe nanocrystals, blade coating, large area fabrication

## Abstract

CdTe semiconductor nanocrystal (NC) solar cells have attracted much attention in recent year due to their low-cost solution fabrication process. However, there are still few reports about the fabrication of large area NC solar cells under ambient conditions. Aiming to push CdTe NC solar cells one step forward to the industry, this study used a novel blade coating technique to fabricate CdTe NC solar cells with different areas (0.16, 0.3, 0.5 cm^2^) under ambient conditions. By optimizing the deposition parameters of the CdTe NC’s active layer, the power conversion efficiency (PCE) of NC solar cells showed a large improvement. Compared to the conventional spin-coated device, a lower post-treatment temperature is required by blade coated NC solar cells. Under the optimal deposition conditions, the NC solar cells with 0.16, 0.3, and 0.5 cm^2^ areas exhibited PCEs of 3.58, 2.82, and 1.93%, respectively. More importantly, the NC solar cells fabricated via the blading technique showed high stability where almost no efficiency degradation appeared after keeping the devices under ambient conditions for over 18 days. This is promising for low-cost, roll-by-roll, and large area industrial fabrication.

## 1. Introduction

Recently, semiconductor nanocrystals (NCs), such as CdTe or PbS NCs, have attracted much attention due to their potential application in photovoltaics [1,2,3,4,5,6,7,8,9], light-emitting diodes [10,11], photodetectors [12,13] or biosensors [14,15] and so on. Compared to other semiconductor NCs, CdTe NCs possess ideal bandgap (~1.5 eV), a high optical absorption coefficient (>10^4^/cm in the visible light region), and high stability, which makes them suitable for photovoltaic application [16]. In addition, CdTe NC solar cells can be fabricated through a simple solution process under ambient conditions. Since 2005, CdTe NC solar cells have been developed rapidly, and a power conversion efficiency (PCE) of up to 12% has been realized by optimizing device architecture and fabricating techniques [17,18,19]. The stability of CdTe NC solar cells with conventional structure, however, remains a challenge because of the misalignment of the work function between ITO (Indium Tin Oxide~4.7 eV) and the CdTe NC (~5.2 eV) thin film [20]. CdTe NC solar cells with inverted structure have therefore received intensive attention as the holes transfer layer can be easily applied in this case for efficient carrier collecting. In previous reports, back-contact layers like MoOx, Cr, etc., had been used to improve carrier collection efficiency in CdTe NC solar cells [21,22,23]. Although the open-circuit voltage (*V*_oc_) had been improved, the PCE of devices was limited due to the non-ideal device designed. Later on, solution-processed organic hole transfer materials (HTMs) such as Spiro-OMeTAD (2,2,7,7-tetrakis (*N,N*-di-4-methoxyphenylamino)-9,9-spirobitluorene) [24] and P3KT (poly-[3-(potassium-6-hexanoate) thiophene-2, 5-diy]) [25] were applied to CdTe NC solar cells, and such devices exhibited a PCE exceeding 6%. Researchers also found that a dipole layer was formed between the CdTe NC and the holes transfer layer (HTL), which reinforced the built-in electric field and improved carrier collection efficiency. Following this, cross-linkable conjugated polymer poly (diphenylsilane-co-4-vinyl-triphenylamine) (Si-TPA) and poly(phenylphosphine-co-4-vinyl-triphenylamine) (P-TPA) were recently presented as HTLs for CdTe NC solar cells [26,27]. Compared to other organic HTLs, cross-linkable conjugated polymer possesses many merits such as high stability, easily adjustable highest occupied molecular orbital (HOMO) and lowest unoccupied molecular orbital (LUMO) levels, and excellent adhesion to the NC thin film surface. By optimizing the thickness and annealing temperature of these HTLs, a notable PCE of ~9% was obtained, which was the highest PCE ever reported for CdTe NC solar cells with inverted structure. However, the NC solar cells listed above are fabricated by the spin-coating method, which is limited to lab-scale, and exhibit high material consumption as spin-coated thin film has poor uniformity over a large area and most of the NCs have been removed during the spin-casting process. Therefore, a new scaled-up fabricating method for large areas must be developed to meet the requirements of industrial mass production of NC solar cells.

Spray coating, slot-die coating, offset coating, gravure coating, and inkjet printing methods have been well developed in recent years for scaled-up manufacturing of thin film solar cells [28,29,30,31]. In 2011, Gianpaolo et al. [32] developed airbrush coating technicques for polymer solar cells. A PCE of 4.1% was obtained in poly (3-hexylthiophene): [6,6]-Phenyl C61 butyric acid methyl-ester (P3HT:PCBM) blend solar cells after optimizing the parameters of the spray system. Later on, Lim et al. [33] presented a new way for polymer solar cells with large areas (~10 cm^2^) to be processed with non-halogenated solvents in air via a blade coating method. Modular devices with a high PCE of ~5% were obtained in this case via optimizing the fabricating parameters. In 2017, Hou and coworkers [34] reported a high PCE of 10.6% on fullerene-free polymer solar cells with a 1 cm^2^ active area using the blade coating method. Poly(2,6′-4,8-di(5-ethylhexylthienyl)benzo1,2-b, 3,3-bdithiophene{3-fluoro-2(2-ethylhexyl)carbonylthieno3,4-bthiophenediyl})(PTB7-th), Poly[(5,6-difluoro-2,1,3-benzothiadiazole-4,7-diyl)[3,3″′-bis(2-octyldodecyl)[2,2′:5′,2″:5″,2″′-quaterthiophene]-5,5″′-diyl]] (PffBT4T-2OD), and other donor polymers with different properties were also developed successfully for large area organic solar cells [35,36]. Most of them delivered a higher PCE of up to 10% and exhibited low efficiency losses, which is important for their industrial applications. In addition to organic solar cells, perovskite solar cells (PSCs), a new type of thin film solar cell, have attracted much attention in recent year due to their cheap ingredients, simple fabricating process, and ultrahigh efficiency (>24%) [37]. In the case of large area devices, Microquanta Semiconductor (China) presented a large area PSC on a rigid substrate with a PCE of 17.4% [38]. Recently, WonderSolar launched a 110 m^2^ photovoltaic (PV) system using large area screen-printed triple mesoscopic PSCs [39]. For PSC large area modules, uniformity of the thin film, pinholes in the active layer, toxicity of the solvents, and stability are all drawbacks that limit their further application [40,41]. In the case of NC solar cells, Matthew et al. [42] first proposed PbS quantum dot solar cells using the solar paint approach and obtained an efficiency of >1%. Although the value is obviously lower than that prepared by the successive ionic layer adsorption and reaction (SILAR)-based method, this solar paint method offers the opportunity to prepare the active layer in a short time, which is preferable for large area fabrication. Later, Kramer et al. [43] developed a room temperature spray coating technique for PbS colloidal quantum dot (CQD) solar cells. In this case, the PbS CQD solution was automatically sprayed on the substrate under computer control and a high PCE of 8.1% was obtained in the final device they made, which implies that there is no compromise between large area manufacturability and lab level. Up to now, printed PbS CQD solar cells with a PCE >10% have been fabricated by modifying PbS colloid ink composition and optimizing deposition methods, which paved the way to commercial application of CQD solar cells [44].

Although the scaled-up fabrication method has been successfully applied in PbS CQD solar cells, there are still few reports on these methods for the fabrication of CdTe NC solar cells. In this work, we develop a novel blade coating method for fabricating CdTe NC solar cells with a device structure of ITO/ZnO/CdSe/CdTe/Au, which allows depositing NC thin films with different areas (0.16, 0.3, and 0.5 cm^2^) fabricated under ambient conditions with low material consumption. The effects of NC ink concentration, active layer thickness, annealing temperature, and blading parameter on the solar cell performance is investigated and discussed in detail. By optimizing the device fabrication parameters, PCEs of 3.58, 2.83, and 1.93% can be obtained for NC solar cells with areas of 0.16, 0.3, and 0.5 cm^2^, respectively. Stability characterization shows that all the NC solar cells with different areas are extremely stable when stored under ambient conditions, with less than 5% degradation for 18 days storage. As all the active layers can be prepared by this simple blade coating method with low material consumption, this work demonstrates a potential way to realize industrial mass production of low-cost NC solar cells.

## 2. Device Fabrication

**Figure 1:**
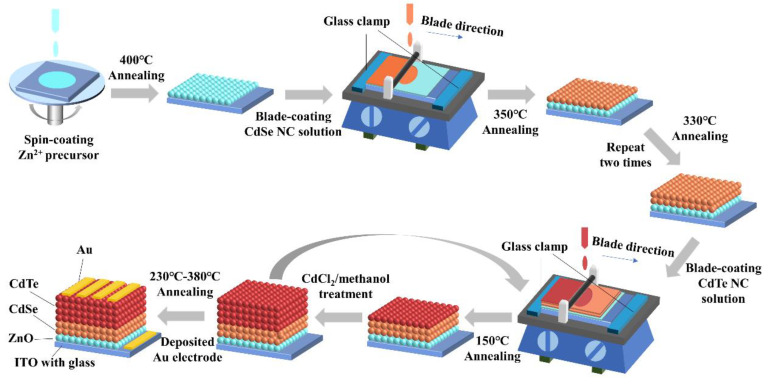
A schematic of the nanocrystal (NC) solar cells fabricated via blade coating.

The Zn^2+^ precursor, CdSe and CdTe NCs were prepared based on the methods reported in [26]. The CdSe NCs were dissolved in pyridine with a concentration of 30 mg/mL, while CdTe NCs were dispersed into a pyridine/n-propanol (with volume ratio of 1:1) solvent with a concentration of 40 mg/mL. In the first step, the ZnO thin film with a thickness of ~40 nm was prepared by spin casting Zn^2+^ precursor on ITO substrate followed by an annealing process of 400 °C for 10 min, as described in [27]. After that, CdSe and CdTe NC films were deposited on the ITO/ZnO substrate in sequence by the blade coating method, as shown in Figure 1. During the blade coating process, several drops of CdSe NC solution were put onto the ITO/ZnO substrate and blade coated evenly using a 10-micron wire rod at a speed of 150 mm/min. Then the substrate was transferred to a hot plate and annealed at 150 °C for 10 min and 350 °C for 40 s to eliminate any organic solvent and impurities. Two or more layers of CdSe NC thin film were fabricated by repeating the above process. The thickness of each layer was about 45 nm. The ITO/ZnO/CdSe substrates were then soaked in methanol and washed ultrasonically. After drying in a N_2_ flow, the CdTe NC thin film was then blade coated on the ITO/ZnO/CdSe substrate at a speed of 150 mm/min. The sample ITO/ZnO/CdSe/CdTe was then placed on a hotplate at 150 °C for 3 min, followed by immediate dipping into saturated CdCl_2_/methanol solution for 10 s and rinsed in 1-propanol for 3 s to remove excess CdCl_2_. The sample was then placed on a hot plate at 350 °C for 40 s and cooled down to room temperature. This process was repeated several times to obtain CdTe NC thin films with different thicknesses (Figure S1 shows the blade coating device). The thickness of each single layer of the CdTe NC was around 110 nm. Finally, the samples were dipped into a saturated CdCl_2_/methanol solution for 3 s and placed on a hot plate at 230~380 °C for 25 min to increase the crystallinity of the CdTe NC thin film. After cleaning in methanol solution, 80 nm Au was then deposited on the ITO/ZnO/CdSe/CdTe through a shadow mask by vacuum thermal evaporation. The final solar cells with areas of 0.16, 0.3, and 0.5 cm^2^ were achieved by using different shadow masks (Figure S2).

## 3. Results and Discussion

**Figure 2:**
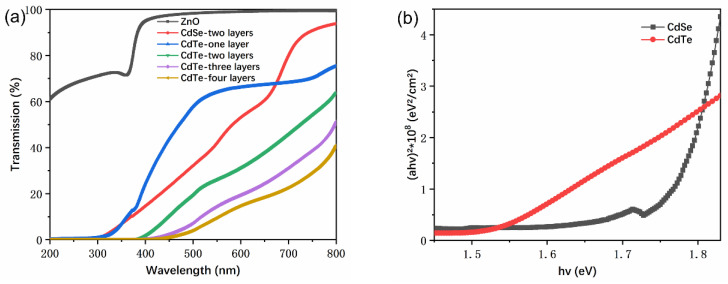
(**a**) Transmission spectra of different ZnO, CdSe NC thin films and CdTe NC thin films with different thicknesses; (**b**) the plots of (*αhv*)^2^ versus photon energy (*hv*) of the CdSe and CdTe NC thin films.

To study the optical properties of the CdTe NC thin films, different thicknesses of CdTe NC thin films were deposited by blade coating on the glass substrate. The transmission spectra for glass/ZnO, glass/CdSe, and glass/CdTe with different thicknesses are showed in Figure 2a. It should be noted that ZnO thin films were transparent from 400 to 800 nm, while significant absorption from 400 to 800 nm was found for the CdTe film with the thickness increase from 110 to 440 nm. From the (*αhv*)^2^ (*α* is the absorption coefficient obtained in Figure 2a) versus photon energy (*hv*) plots (Figure 2b), it is obvious that all samples had a linear onset, indicating a direct bandgap for CdSe and CdTe NC thin films. The bandgaps of CdSe and CdTe NC thin films were 1.78 eV and 1.51 eV, respectively, which was calculated by extrapolating the linear region of this plot to the *x*-axis.

**Figure 3:**
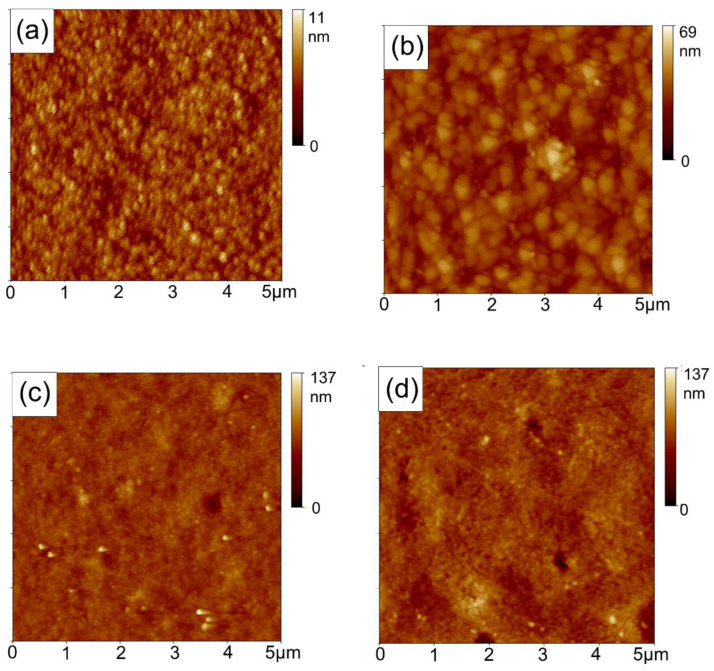

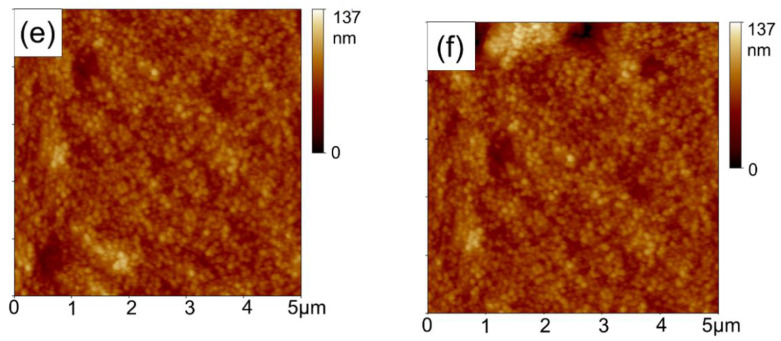
Atomic force microscopy (AFM) images of (**a**) ITO/ZnO thin film; (**b**) ITO/ZnO/CdSe thin film; and ITO/ZnO/CdSe/CdTe with annealing temperatures of (**c**) 250; (**d**) 290; (**e**) 310; and (**f**) 380 °C.

The morphologies of ITO/ZnO, ITO/ZnO/CdSe, and ITO/ZnO/CdSe/CdTe with different annealing temperatures are presented in Figure 3. It is obvious that the ZnO and CdSe NC thin films were very smooth and compact with root mean square (RMS) roughness values of 1.03 and 5.46 nm, respectively (Figure 3a,b). The morphology of the CdTe NC thin film was affected by the annealing temperature. The RMS roughness value of the CdTe NC films with annealing temperatures of 250, 290, 310, and 380 °C were 6.74, 9.39, 10.5, and 13.1 nm, respectively (Figure 3c–f). This suggests that the blade coated CdTe NC thin films are homogeneous and annealing temperature does not have a significant influence on the roughness of the thin film. A smooth CdTe NC thin film is essential to eliminate interface recombination and increase carrier collection efficiency. Besides, it should be noted that there are some pinholes for the NC samples treated under high annealing temperature (Figure 3f), which may result in large leakage current. From this point of view, a high annealing temperature is not recommended for the post-treatment of our NC solar cells.

**Figure 4:**
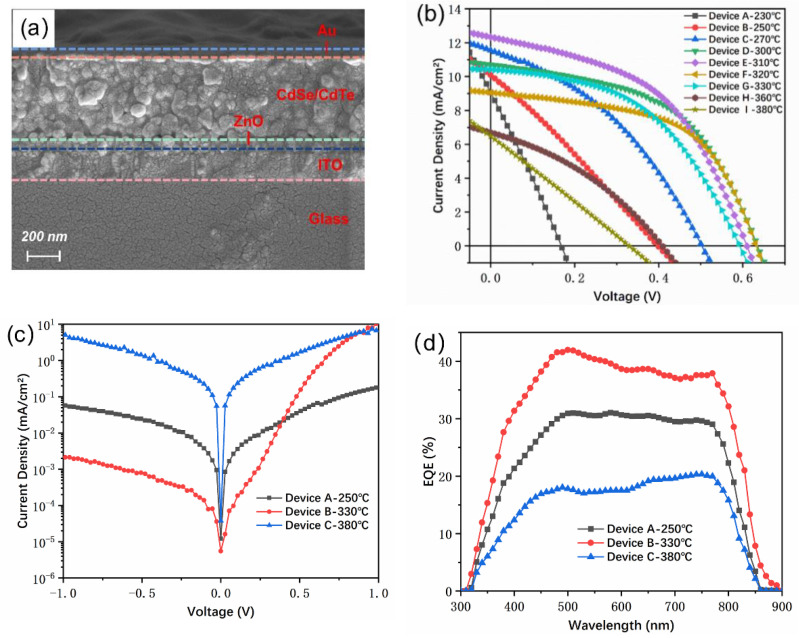
(**a**) Cross-section SEM images of ITO/ZnO/CdSe/CdTe/Au; J–V curves of ITO/ZnO/CdSe/CdTe/Au with different annealing temperatures (**b**) under light and (**c**) under dark; (**d**) the corresponding external quantum efficiency (EQE) spectra.

A sintering process could help promote the growth of CdTe crystal grains, eliminate defects inside the thin film or at the interface, and increase the carrier concentration effectively. Therefore, the annealing temperature is crucial to improve the device performance of solution-processed CdTe NC solar cells, which was confirmed in a previous work [8]. In those reports, a moderate temperature of 350 °C was chosen to be the best annealing temperature for CdTe NC solar cells. Devices fabricated via blade coating, however, are quite different from the spin-coated ones, and the best annealing temperature might be different in this case. To investigate the effects of annealing temperature on device performance, the thickness of the CdTe NC was fixed at ~400 nm, prepared by depositing four layers of CdTe NC on the ITO/ZnO/CdSe substrate. The ITO/ZnO/CdSe/CdTe was treated with CdCl_2_ in methanol and then annealed at 230~380 °C under ambient conditions. Figure 4a shows the cross-section SEM images of an NC device with a configuration of ITO/ZnO/CdSe/CdTe/Au. The CdTe/CdSe NC layer was compact with thickness of ~600 nm, which consisted of two layers of CdSe NC and four layers of CdTe NC. Figure 4b presents the J–V curves of CdTe NC solar cells with different annealing temperatures under 1000 W m^−2^ (AM 1.5 G), while the device performance parameters are summarized in Table 1. It is noted that although the short-circuit current density (*J*_sc_ = 10.09 mA/cm^2^) was high when annealed under a low temperature of 250 °C, the device had a low *V*_oc_ (0.39 V) and fill factor (FF = 27.49%), which resulted in a low PCE of 1.08%. When the annealing temperature increased to the range of 290~330 °C, a PCE up to 3% was obtained, which was mainly attributed to the increase of *V*_oc_. The champion device was obtained at an annealing temperature of 310 °C, the *J*_sc_, *V*_oc_, and FF were 12.33 mA/cm^2^, 0.60 V, and 48.38% respectively, delivering a relatively high PCE of 3.58%. On the other hand, when the annealing temperature was further increased from 330 to 380 °C, the PCE dropped down linearly and only a 0.54% PCE was obtained at 380 °C. All the device parameters (*J*_sc_, *V*_oc_ and FF) deteriorated at a relatively high annealing temperature (>350 °C). The changes in devices performance with different annealing temperatures can be explained from multiple perspectives. First of all, under low annealing temperature, the diffusion of Cl^−^ is inadequate, leading to low hole concentration in the CdTe NC thin film. Such low hole concentration might affect the Fermi level of the *p*-type region, resulting in a low built-in electric field in the space charge region. In this case, a low *V*_oc_ and low device performance are expected. On the other hand, if the annealing temperature is too high (>360 °C), the quantity of pinholes or metal oxide may increase inside the CdTe NC thin film or at the interface, which will dramatically deteriorate the device performance. From Table 1, one can see that the device shunt resistance (*R*_*sh*_) is below 140 Ω, implying large leakage current and low *p*–*n* junction quality at high temperature. Similar results can also be found in our previous report on CdTe NC solar cells with inverted structure [8]. When the annealing temperature was in the range of 290 to 330 °C, all the devices showed high *V*_oc_ (>0.6 V) and FF (>40%), leading to a PCE up to 3%. In these cases, the series resistance (*R*_*s*_) was in the range of 10~20 Ω·cm^2^ and *R*_*sh*_ was higher than 200 Ω·cm^2^, indicating high *p*–*n* junction quality. The typical dark J–V curves of devices annealing at 250, 330, and 380 °C are presented in Figure 4c. Obviously, for device treatment at 330 °C, the dark current at reverse bias voltage was significantly lower than the device at 250 or 380 °C. Therefore, the optimized annealing temperature for blade coated CdTe NC solar cells is ~300 °C. Figure 4d presents the typical external quantum efficiency (EQE) spectra of devices, the EQE value was around 40 between 450 to 900 nm. When EQEs are integrated, *J*_sc_ of 8.22, 10.83, and 5.10 mA/cm^2^ are predicted for devices annealed at 250, 330, and 380 °C respectively, which are consistent with the J–V curves presented in Figure 4b. The *J*_sc_ (~10 mA/cm^2^) value of NC devices prepared by blade coating was lower than that (~20 mA/cm^2^) for devices prepared by the spin-coating method with similar device architecture [8], which may attributed to the difference in CdTe and CdSe NC film quality. The NC solar cells with different thicknesses of CdTe NC films treated under optimized sintering conditions were also investigated, and a low PCE was found for CdTe NCs with three or five layers (as shown in Figure S3).

**Figure 5:**
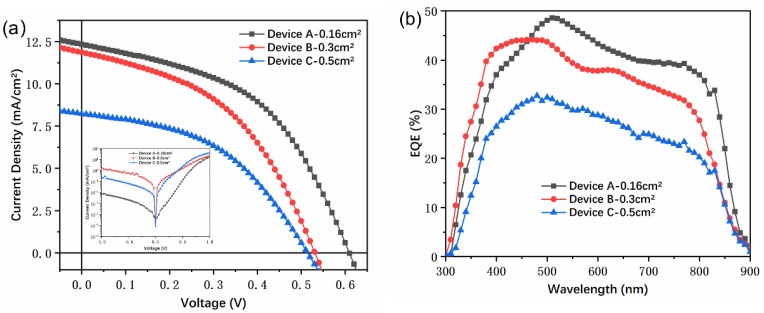
(**a**) J–V curves of ITO/ZnO/CdSe/CdTe/Au with different active areas (Device A—0.16 cm^2^, Device B—0.3 cm^2^, Device C—0.5 cm^2^) under light (inset is J–V under dark); (**b**) the corresponding external quantum efficiency (EQE) spectrum.

The CdTe NC thin film was proved to be extremely stable and could be fabricated under ambient conditions. To ascertain the performance of NC solar cells with different areas, devices with an inverted structure of ITO/ZnO (40 nm)/CdSe (90 nm)/CdTe (400 nm)/Au (80 nm) were fabricated by blade coating four CdTe NC layers on CdSe film. Devices with 0.3 and 0.5 cm^2^ were fabricated by depositing a contact metal electrode through shadow masks with active areas of 0.3 and 0.5 cm^2^ (as shown in Figure S2). For the device with an active area of 0.3 cm^2^, a short-circuit current density (*J*_sc_) of 11.86 mA/cm^2^, an open-circuit voltage (*V*_oc_) of 0.53 V, a fill factor (FF) of 44.77%, and a PCE of 2.82% were obtained, while these value for 0.5 cm^2^ were 8.23 mA/cm^2^, 0.51 V, 45.38%, and 1.93%, respectively (Figure 5a and Table 2). The device performance degraded as the active area increased, which is primarily ascribed to the reduced in *V*_oc_. For the 0.16 cm^2^ device the *V*_oc_ was 0.6 V, while this value was 0.53 and 0.51 V for 0.3 and 0.5 cm^2^ devices, respectively. We speculate that the defects in the CdSe and CdTe NC thin films increased when the active area increased. Moreover, the junction quality may have dropped down due to the homogeneity decrease in a large area. Finally, the series resistance of ITO and interfacial contact resistance would increase with increasing active area. All these factors listed above resulted in low device performance for large area devices, and similar results were also reported in the case of PSC [39]. It is noted that the pattern ITO substrate with an active area of 1.5 cm × 1 cm (Length × width) was used here. However, the 0.5 cm^2^ device showed length × width = 0.63 cm × 0.8 cm (Figure S2), and the width of the NC devices was close to that of ITO substrate. In this case, the boundary effects may increase defects and decrease device performance. To further investigate the drying process on the large area NC devices (0.5 cm^2^) performance, devices with different annealing temperature are presented. The J–V curves are presented in Figure S4 and the solar cell parameters are summarized in Table S1. NC solar cells showed an improved PCE of 2.11% in the case of 320 °C annealing temperature. The annealing temperature affects the drying process of NC thin film, high quality NC thin film can be formed at optimized annealing temperature. The selected solvent may also affect the drying process and the device performance; more work and design experiment will be carried out in our subsequent research. We believe that the PCE of NC solar cells with large areas can certainly be improved via designing and using a suitable pattern ITO substrate. Figure 5b shows the EQE curves of devices with different areas. It should be noted that all the devices show similar shape at the whole short wavelength. The device with a large area prepared by blade coating was not further tuned in this paper, and we believe that there may be room for further improvement.

**Figure 6:**
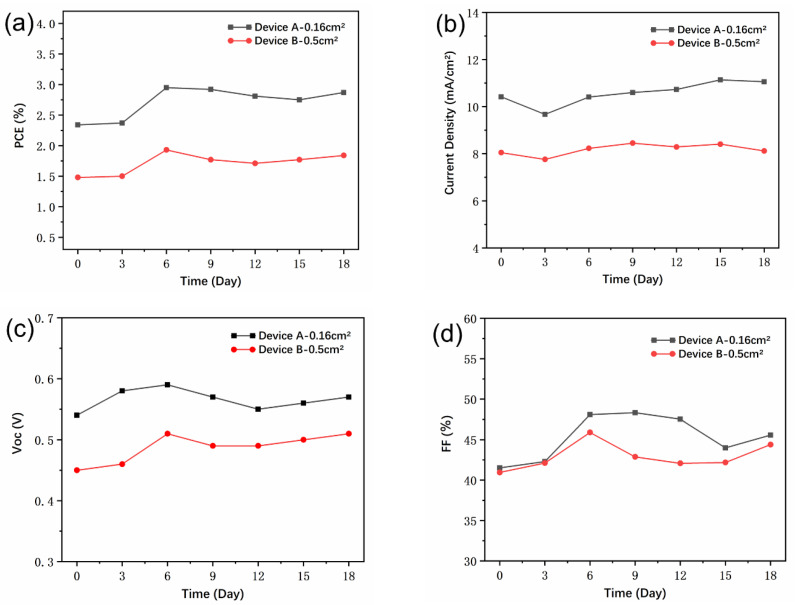
Evolution of parameters (**a**) PCE (power conversion efficiency), (**b**) $J_{\mathrm{sc}}$, (**c**) $V_{\mathrm{oc}}$, and (**d**) FF for devices with different active areas stored under ambient conditions (Device A—0.16 cm^2^, Device B—0.5 cm^2^).

One challenge for commercial application is the stability of NC solar cells. For CdTe NC solar cells, the interfacial layer, the grain boundaries, the contact electrodes, and the diffusion of dopant elements are the main sources of instability. In the previous report, we found that CdTe NC solar cells with inverted structure (ITO/ZnO/CdSe/CdTe/Au) were very stable, with negligible change in PCE when stored under ambient conditions [45]. As the post-treatment process and the device structure in this research are similar to those devices previously reported, blade coated NC solar cells prepared the same way should have similar stability. Here, the long-term operation stability of CdTe NC solar cells with different areas is presented in Figure 6. The *V*_oc_, *J*_sc_, FF, and PCE of NC solar cells increased in the first several days. As shown in Figure 6a, the PCE for original devices with areas of 0.16 and 0.5 cm^2^ were 2.34 and 1.48%, respectively, while these values were 2.95 and 1.93%, respectively on Day 6. From the *J*_sc_, *V*_oc_, and FF curves of NC devices (Figure 6b–d), one can see that the improvement in device performance was mainly attributed to the increase in *V*_oc_ and FF. The performance enhancement after storage was found in the case of PbS quantum dot solar cells and CdTe NC solar cells [46,47]. We speculate that the diffusion between CdTe and CdSe NC thin films and the Au on the CdTe NC interface with storage time will eliminate some interface defects, which will increase the carrier collecting efficiency and improve device performance. It should be noted that the NC devices prepared by blade coating showed outstanding stability and less than 5% degradation in PCE after storage at ambient conditions for 18 days.

In conclusion, we developed a blade coating method for fabricating CdTe NC solar cells with large areas. We found that the annealing temperature has significant influence on the NC solar cells. The optimized annealing temperature for CdTe NC solar cells is in the range of 300 to 330 °C, which is different from those NC solar cells prepared by the spin-coating method. At an annealing temperature of 310 °C, a champion device shows a *J*_sc_ of 12.33 mA/cm^2^, a *V*_oc_ of 0.60 V, an FF of 48.38%, delivering a PCE of 3.58%. It should be noted that NC solar cells with large areas of 0.3 and 0.5 cm^2^ can also be easily prepared through the blade coating process and only a small decrease in PCE is found, implying the scalability of this method. The NC solar cells show good stability and less than 5% degradation is found when the device is stored under ambient condition for 18 days. The exploration in this work provides valuable suggestions and solutions for low-cost and large-scale fabricating of NC solar cells.

## Figures and Tables

**Table 1:** Summarized performance of NC solar cells with different annealing temperatures (Figure 4b).

Device	Temperature (°C)	*V*_oc_ (V)	*J*_sc_ (mA/cm^2^)	FF (%)	PCE (%)	$R_{s}$ $\;(\Omega \cdot cm^{2})$	$R_{sh}$ $\;(\Omega \cdot cm^{2})$
A	230	0.16	9.06	27.98	0.41	16.62	20.60
B	250	0.39	10.09	27.49	1.08	38.57	50.37
C	270	0.50	11.55	37.97	2.19	21.29	111.19
D	300	0.63	10.70	51.34	3.46	15.89	351.93
E	310	0.60	12.33	48.38	3.58	15.97	201.59
F	320	0.63	9.05	55.66	3.17	15.34	357.88
G	330	0.59	10.41	48.06	2.95	18.72	694.60
H	360	0.40	6.70	35.77	0.96	32.99	137.42
I	380	0.32	6.47	26.26	0.54	49.24	54.70

**Table 2:** Summarized performance of NC solar cells with different active areas (Figure 5).

Device	Active Area (cm^2^)	*V*_oc_ (V)	*J*_sc_ (mA/cm^2^)	FF (%)	PCE (%)	$R_{s}$ $\;(\Omega \cdot cm^{2})$	$R_{sh}$ $\;(\Omega \cdot cm^{2})$
A	0.16	0.60	12.33	48.38	3.58	15.97	201.59
B	0.3	0.53	11.86	44.77	2.82	15.42	175.56
C	0.5	0.51	8.23	45.38	1.93	23.82	313.39

## Data Availability

Data is contained within the article or Supplementary Material.

## Supplementary Materials

The following are available online at https://www.mdpi.com/article/10.3390/nano11061522/s1, Figure S1. Equipment for doctor-blading preparation devices, Figure S2. Devices with different active areas (**a**) 0.16 cm^2^; (**b**) 0.3 cm^2^; (**c**) 0.5 cm^2^, Figure S3. J–V curves of ITO/ZnO/CdSe/CdTe/Au with different CdTe layers at 310 °C (**a**) three layers (**b**) five layers, Figure S4. J–V curves of ITO/ZnO/CdSe/CdTe/Au at different annealing temperatures (Device A: 320 °C, Device B: 340 °C, Device C: 360 °C) under light with 0.5 cm^2^ active area.
