# Supplementary material for: Efficient Nanocrystal Photovoltaics via Blade Coating Active Layer
Source: Nanomaterials (Basel). 2021 Jun 9;11(6):1522. doi: 10.3390/nano11061522 (PMC8226763; doi:10.3390/nano11061522)
Supplement: Supplementary file 1 [file nanomaterials-11-01522-s001.zip › nanomaterials-1235451-supplementary.pdf]

nanomaterials

Supplementary Materials: Efficient Nanocrystal Photovoltaics via Blade Coating Active Layer

Kening Xiao [1], Qichuan Huang [1], Jia Luo [1], Huansong Tang [1], Ao Xu [1], Pu Wang [1], Hao Ren [1], Donghuan Qin [1,2,*], Wei Xu [1,2,*] and Dan Wang [1,2,*]

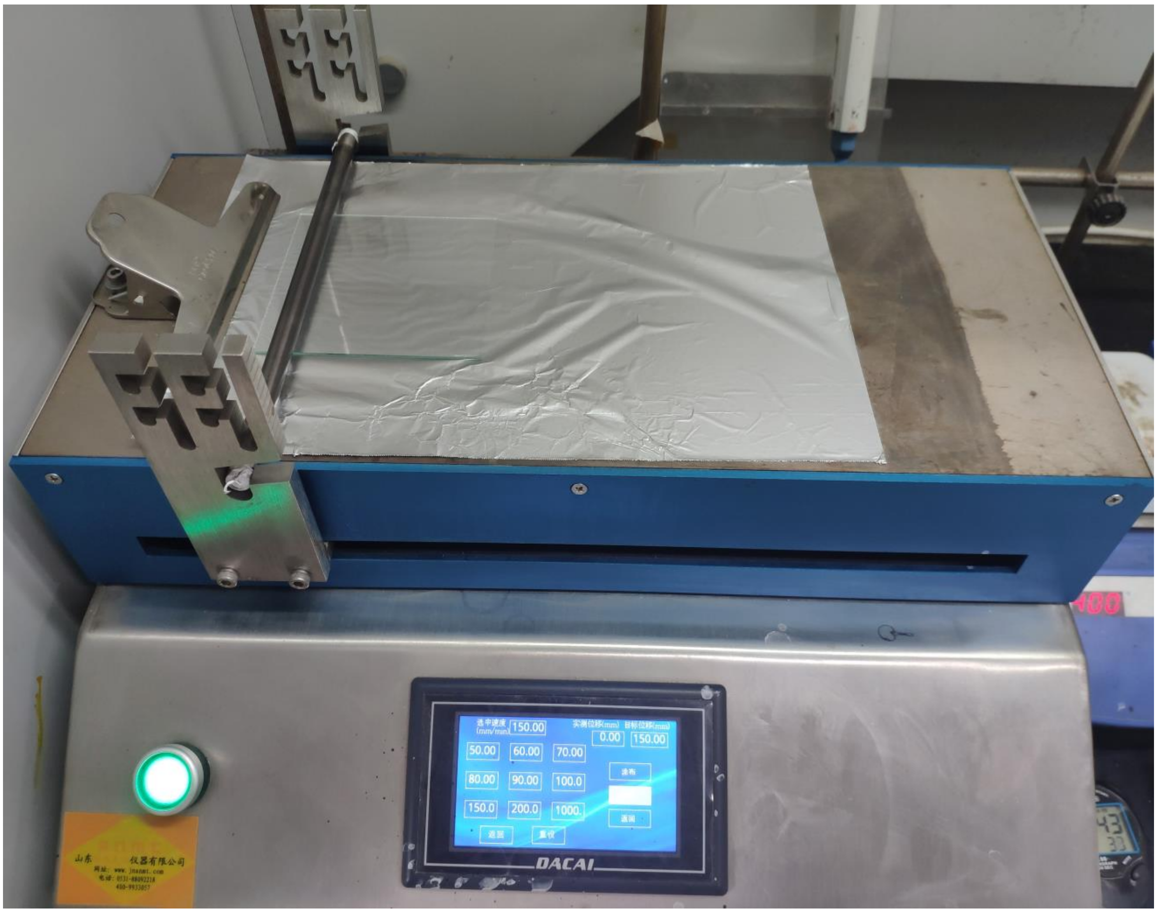

Figure S1. Equipment for doctor-blading preparation devices.

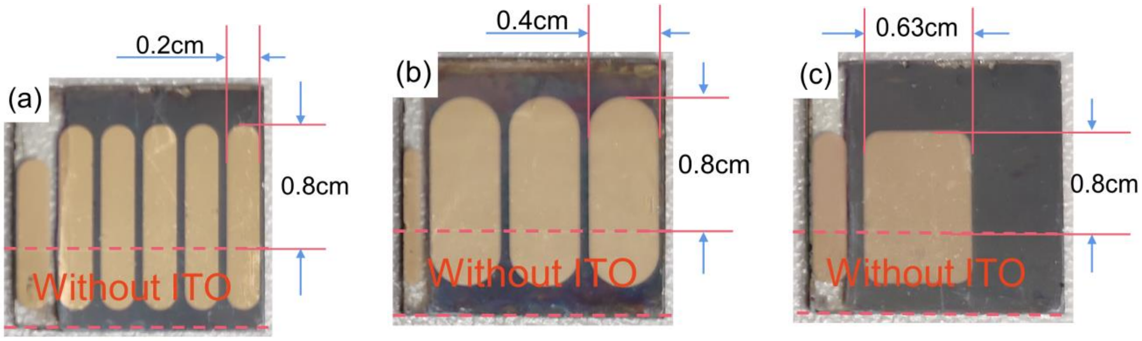

Figure S2. Devices with different active areas (**a**) 0.16 cm²; (**b**) 0.3 cm²; (**c**) 0.5 cm².

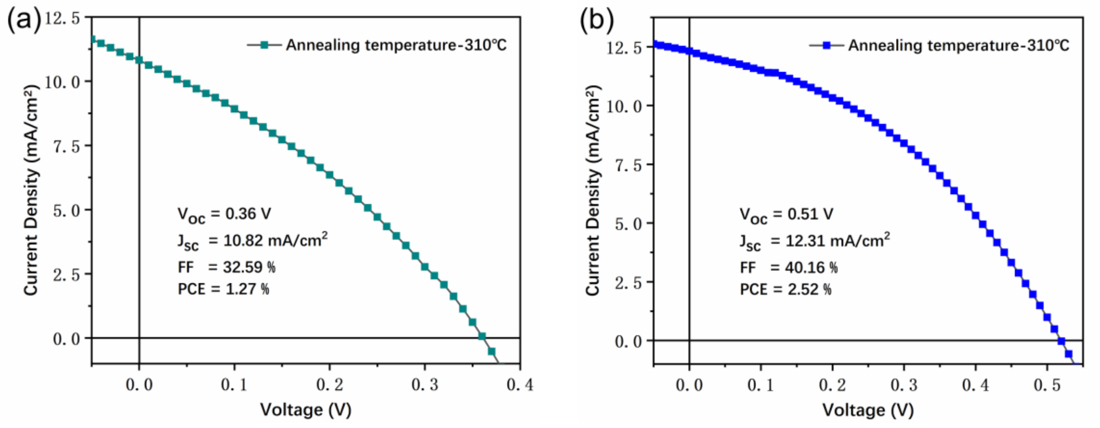

Figure S3. J–V curves of ITO/ZnO/CdSe/CdTe/Au with different CdTe layers at 310 °C (**a**) three layers (**b**) five layers.

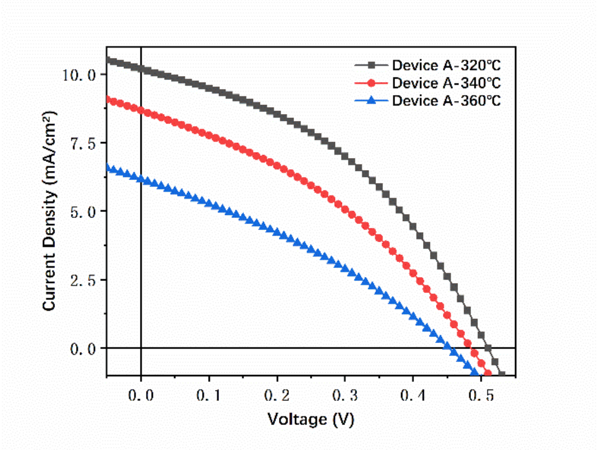

Figure S4. J–V curves of ITO/ZnO/CdSe/CdTe/Au at different annealing temperatures (Device A:320 °C, Device B:340 °C, Device C:360 °C) under light with 0.5 cm^2 active area.

Table S1. Summarized performance of NC solar cells with different annealing temperatures (Figure S4).

Device	Temperature (℃)	V_{oc} (V)	J_{sc} (mA/cm^2)	FF (%)	PCE (%)
**A**	**320**	0.50	10.19	41.40	2.11
**B**	**340**	0.48	8.67	36.59	1.52
**C**	**360**	0.45	6.16	32.39	0.90

Equipment information

The cross-section diagram was obtained from Zeiss Sigma300's scanning electron microscope (SEM). The atomic force microscopy (AFM) imagines were obtained using a NanoScope NS3A system (Veeko, CA, USA). The external quantum efficiency (EQE) was measured by a Zolix instrument (Solar Cell Scan100, Zolix Instruments Co., Ltd., Beijing, China). The J–V characteristics were measured with a Keithley 2400 under an illumination of 100 mW/cm^2 with an air mass 1.5 (AM 1.5) solar simulator (Oriel model 91192).
